# For Whom Does Mindfulness-Based Stress Reduction Work? Moderating Effects of Personality

**DOI:** 10.1007/s12671-017-0687-0

**Published:** 2017-02-15

**Authors:** Ivan Nyklíček, Mona Irrmischer

**Affiliations:** 1 Center of Research on Psychology in Somatic diseases (CoRPS), Department of Medical and Clinical Psychology, Tilburg University, P.O. Box: 90153, NL-5000 Tilburg, LE Netherlands; 2 Center for Neurogenomics and Cognitive Research, Department of Integrative Neurophysiology, Vrije University, Amsterdam, Netherlands

**Keywords:** Anxiety, Depression, Intervention, Mindfulness, Moderator, Personality

## Abstract

The aim of the present study was to examine potentially moderating effects of personality characteristics regarding changes in anxious and depressed mood associated with Mindfulness-Based Stress Reduction (MBSR), controlling for socio-demographic factors. Meditation-naïve participants from the general population self-presenting with psychological stress complaints (*n* = 167 participants, 70% women, mean age 45.8 ± 9.3 years) were assessed in a longitudinal investigation of change in mood before and after the intervention and at a 3-month follow-up. Participants initially scoring high on neuroticism showed stronger decreases in both anxious and depressed mood (both *p* < 0.001). However, when controlled for baseline mood, only the time by neuroticism interaction effect on anxiety remained significant (*p* = 0.001), reflecting a smaller decrease in anxiety between pre- and post-intervention but a larger decrease in anxiety between post-intervention and follow-up in those with higher baseline neuroticism scores. Most personality factors did not show moderating effects, when controlled for baseline mood. Only neuroticism showed to be associated with delayed benefit. Results are discussed in the context of findings from similar research using more traditional cognitive-behavioral interventions.

## Introduction

Mindfulness-Based Stress Reduction (MBSR) (Kabat-Zinn 1990; Kabat-Zinn et al. 1992) is a widely applied and frequently researched psychological intervention, which is aimed at the cultivation of a mindful attitude. This attitude, consisting of a set of learnable skills, is often defined as being attentive to phenomena occurring in the present moment in a nonjudgmental or accepting way (Baer et al. 2006; Brown and Ryan 2003; Kabat-Zinn 1990). Recent systematic reviews (Chiesa and Serretti 2010; Fjorback et al. 2011; Keng et al. 2011) and meta-analyses (Hofmann et al. 2010; Khoury et al. 2013) on the effectiveness of MBSR regarding psychological outcomes have concluded that MBSR and its variant Mindfulness-Based Cognitive Therapy (MBCT) (Segal et al. 2002) are effective regarding the reduction of psychological symptoms of distress like anxious and depressed mood. When compared to waitlist or treatment-as-usual control groups, on average, a medium effect size was reported (Hofmann et al. 2010), while these interventions are about as effective as cognitive behavioral therapy (Khoury et al. 2013).

Even though MBSR is a treatment for which specific person characteristics are claimed to be important, such as high personal commitment and openness to new experiences (Kabat-Zinn 1990), little emphasis has been put on research into possible moderating influence of personality traits of participants to examine for whom this form of intervention might be most (and least) beneficial. The identification of a possible moderator that might influence the direction or magnitude of the effects of the intervention on outcome is important from both theoretical and clinical perspectives.

From a theoretical perspective, the identification of factors moderating the effects may inform theory building regarding the working mechanisms of mindfulness; when we know for whom it works better, this may suggest possible mechanisms of action. Various mechanisms have been proposed in the literature, which are mindfulness processes such as acceptance and nonreacting to inner phenomena (Baer et al. 2006), decentering/cognitive defusion leading to cognitive and behavioral flexibility (Hayes and Feldman 2004; Shapiro et al. 2006), enhancement of emotion regulation skills (Chambers et al. 2009; Nyklíček 2011), and decrease of rumination and worry (Baer 2009; Segal et al. 2002). A recent systematic review of empirical studies on mechanisms of change in MBCT provided support for a mediating effect of several of these constructs (van der Velden et al. 2015).

Some empirical evidence exists regarding moderator effects by pre-treatment mindfulness levels. In a small randomized trial comparing MBSR with a waitlist control group, it was shown that pre-treatment mindfulness was positively associated with increases in psychological well-being (Shapiro et al. 2011). Also regarding another intervention, involving expressive writing, higher initial mindfulness levels seemed to be associated with more psychological benefit (Poon and Danoff-Burg 2011). These results suggest that the mechanism here involves one building on skills already present before the intervention to some extent.

Regarding more fundamental attributes, there is a paucity of studies examining potentially moderating effects of personality characteristics in MBSR and MBCT. Regarding moderation by personality characteristics, to the best of our knowledge, this has not been examined to date for mindfulness-based interventions in patient or general populations. Only a recent controlled study of MBSR in undergraduate and medical students showed that higher neuroticism was associated with a stronger reduction of psychological distress (de Vibe et al. 2015). The authors interpreted the finding as being potentially due to efficacy of learning new emotion regulation skills in these people with a tendency to react strongly emotionally to challenges. This is an example of how identification of a moderator may generate hypotheses regarding working mechanisms of mindfulness, which may be different depending on the moderator involved.

This paucity of research on personality as moderators is peculiar as personality characteristics, such as neuroticism and extraversion, are associated not only with psychological well-being but also with mindfulness skills (Baer et al. 2004; Baer et al. 2006; Brown and Ryan 2003) and (other) putative mechanisms, such as emotion regulation skills (Kokkonen and Pulkkinen 2001), rumination (Trapnell and Campbell 1999), and cognitive flexibility (Kashdan and Rottenberg 2010). Regarding mindfulness skills, a meta-analysis on the general relationship between personality and mindfulness has concluded that the strongest relationship with overall mindfulness was found for neuroticism (*r* = −0.45) and conscientiousness (*r* = 0.32) (Giluk 2009).

Neuroticism has been most often studied in its relation to mindfulness facets, showing especially strong negative correlations not only with the mindful skill of nonjudgmental acceptance but also with acting with awareness and being nonreactive to one’s disturbing thoughts (Baer et al. 2004; Baer et al. 2006; Brown and Ryan 2003; Hanley 2016). In addition, neuroticism is known to be positively associated with a tendency to ruminate (Costa and McCrae 1992) and with avoidant behavior (Lommen et al. 2010), both being mechanisms exacerbating negative mood and both being counteracted by mindfulness (Hayes and Feldman 2004). It may be hypothesized that some of these mechanisms may be involved in increasing emotion regulation skills in individuals scoring high on neuroticism as mentioned above, resulting in stronger effects in these people (de Vibe et al. 2015). Finally, also because negative mood itself is higher in these individuals, more gain may be expected in people scoring high on neuroticism. However, because of this association, analyses should take baseline levels of mood into account to adjust for their effects.

Regarding conscientiousness and mindfulness skills, the strongest correlations were reported with acting with awareness, being a positive association (Baer et al. 2004; Hanley 2016). It is unclear whether less benefit should be expected for high-conscientiousness people because they already are relatively high on this mindfulness facet. More likely, as performing almost daily mindfulness practice is required for participants, the self-disciplined facet of conscientiousness may be hypothesized to contribute to an enhanced benefit, the amount of resulting practice being the driving force potentially increasing any of the mechanisms of action.

Extraversion has been showing rather weak and inconsistent associations with mindfulness (Giluk 2009; Hanley 2016), although it is sometimes reported to be associated with several mindfulness facets (Hollis-Walker and Colosimo 2011). The inconsistencies may stem from the heterogeneity of the construct, including characteristics such as being energetic, gregarious, and adventurous. Theoretically, while gregariousness may benefit participation in a group intervention in general, being adventurous may form a potential misfit with the introspective nature of the intervention. Extraversion as a whole is usually associated with good emotion regulation, low rumination, and positive affect (Costa and McCrae 1992), potentially resulting in lower benefit in individuals high on this personality dimension as a consequence of a ceiling effect in these mechanisms. Conversely, in these areas, gains may be expected, such as enhanced awareness and acceptance of internal phenomena. In addition, the higher psychological flexibility associated with extraversion (Kashdan and Rottenberg 2010) may be expected to facilitate benefits of an intervention calling upon a change in perspective and change in attitude towards mental phenomena, again potentially enhancing mainly acceptance.

Openness has been associated with several mindfulness skills, especially observing and describing (Bohlmeijer et al. 2011; Hollis-Walker and Colosimo 2011), although associations are often not very strong and not always found (Latzman and Masuda 2013). Openness to experience has also been related to greater psychological flexibility (Kashdan and Rottenberg 2010). The potential resulting larger benefit in people scoring high on this trait may be even stronger in the context of a mindfulness-based intervention, as it involves learning an approach very different from conventional modes of thinking.

Agreeableness has shown inconsistent associations with general mindfulness (Giluk 2009). While sometimes it has been related to various mindfulness skills (Baer et al. 2004; Hanley 2016; Hollis-Walker and Colosimo 2011), correlations were stronger with other personality traits, mainly neuroticism. Most importantly, no theoretical grounds were found for expecting a moderating effect of agreeableness. Therefore, this trait was not included in the present study.

Consequently, the aim of the present study was to examine potentially moderating effects of four of the five basic personality characteristics regarding changes in mood associated with MBSR. The moderating influence of personality traits was also examined on the longer term well-being at a 3-month follow-up. Given the rationales discussed above, moderating effects of personality on outcome of MBSR were hypothesized for pre-intervention levels of neuroticism, extraversion, conscientiousness, and openness. All dimensions were hypothesized to be positively associated with favorable changes in mood, reflected by decreases in anxious and depressed mood. Considering the arguments above, strongest effects were hypothesized for neuroticism, openness to experience, and the self-discipline facet of conscientiousness, while for extraversion, the expectations were lowest, rendering the tests on this trait most explorative in nature.

## Method

### Participants

Participants were recruited by means of advertisements in the local newspapers in Tilburg, southern Netherlands. The MBSR intervention was advertised as a group intervention to reduce feelings of distress by means of cultivating mindfulness. By means of a brief online document and questionnaire, (i) people were informed about the training and study and (ii) potential exclusion criteria were checked, which are severe psychiatric disorders (e.g., current severe major depression episode, suicidal, or psychotic tendencies), current psychological treatment, and inability to read or write in Dutch. However, none of the people interested in participation were excluded.

According to sample size calculation (G*Power 3.1.9.2) based on a repeated measure within-between group interactions of a medium effect size, alpha of 0.05, and power of 0.90, a required sample size emerged of 143 participants. In light of an expected attrition of 15% until the 3-month follow-up, we aimed to include 170 participants.

### Procedure

MBSR according to the manualized group program by Jon Kabat-Zinn (1990) was applied. It consists of eight sessions of 2.5 h over 8 to 10 weeks, a silent retreat session of 6 h in the sixth week, and daily homework practice of at least 40 min. The program trains mindfulness by means of meditation practice (e.g., body scan and sitting meditation), yoga, psycho-education (e.g., on automatic versus mindful response), and group sharing of experiences during the sessions. In the mindfulness practices, participants are instructed to observe their experiences, recognize when their attention drifts away, and to redirect their attention to their experiences in a nonjudgmental way. During the study period, 12 groups of 13–15 participants were run by a qualified psychologist who received extensive training in MBSR and is certified by the Dutch Association for Mindfulness (VVM).

Information letter with a consent form was sent to the participants, and the consent form was collected at the first training session. Participants completed online questionnaires before the first meeting. The questionnaires were completed again after completion of the program, 8–10 weeks later, and at a 3-month follow-up after the end of the training.

## Measures

### Socio-Demographic and Health Measures

Socio-demographic variables included in the questionnaire were gender, age, education, occupation, and marital status. Self-reported medical information included previous treatment of psychological disorders (depression, anxiety, general distress, or burnout) and current use of psychotropic medication.

### Personality and Mood Measures

To assess personality, selected facets were administered of the Revised Dutch version of the NEO Personality Inventory (NEO-PI-R) (Costa and McCrae 1992; Hoekstra et al. 1996). This was done to apply the assessment tool in an economical way to (i) use only facets for which moderating effects may be anticipated based on theoretical considerations and empirical findings discussed and (ii) reduce participants’ burden. Only facets from four of five higher-order traits were selected, which are *conscientiousness* (the degree of self-organization), *extraversion* (the degree of outward direction of attention and energy), *neuroticism* (even tempered versus chronic negative affects), and *openness* to experience (curiousness versus narrow mindedness). Agreeableness was not assessed as this trait seemed less relevant in this context, as described in the “Introduction” section. Of these 4 traits, 11 specific facets were selected, from the personality trait neuroticism, the facets N1 (anxiety) and N3 (depression); from extraversion, the facets E2 (gregariousness), E4 (energy), and E5 (adventurism); from the trait openness to experience, O3 (openness to feelings), O4 (openness to actions), and O5 (openness to ideas); and from conscientiousness, the facets C1 (efficacy), C2 (order), and C5 (self-discipline). All facet scales consist of eight items scored on five-point Likert scales, ranging from 1 (strongly disagree) to 5 (strongly agree). Internal consistencies of these subscales are reported to be adequate, with median Cronbach’s alphas between 0.63 (adventurism and openness to change) and 0.83 (anxiety) (Hoekstra et al. 2003). In the present study, all facet scales had a Cronbach’s alpha above *α* = 0.70, except openness for change (*α* = 0.67), and the energy (*α* = 0.68) and adventurism (*α* = 0.67) facets of extraversion. Validity of the original Dutch scales has been established by showing substantial correlations with related constructs and low correlations with unrelated constructs (Hoekstra et al. 2003).

Because we used only selected facets of the higher-order traits, it was desirable to examine the internal validity of these traits. In the Dutch manual, it was already shown that all facets had substantial (all *r* > 0.55) correlations with the corresponding higher-order traits. This may be interpreted as reflecting convergent validity (Carlson and Herdman 2012) with the higher-order trait.

More importantly, we performed a principle component analysis with Varimax rotation using the facet scores as variables, as also reported in the Dutch Manual (Hoekstra et al. 2003). The solution showed the expected four factors with eigenvalues >1.0, explaining 68% of the variance. The first factor, explaining 27.6%, showed very high loadings (>0.80) of only the two Neuroticism facets, with two additional loadings >0.40 by two other facet scales (Table 1). On the three other factors, only each of the three facet scales belonging to the traits loaded >0.40 on the corresponding higher-order trait (>0.69 for conscientiousness, >0.61 for extraversion, and >0.52 for openness). These outcomes, including occasional double loadings, are very similar to the full questionnaire as reported in the Dutch Manual (Hoekstra et al. 2003). Cronbach alpha’s for the resulting higher-order trait scales based on their corresponding facet scale items were 0.92 (neuroticism), 0.81 (extraversion), 0.85 (conscientiousness), and 0.78 (openness). These figures, as well as the Cronbach alpha’s of the facet scales, were also highly comparable to those reported in the Dutch Manual for the full NEO-PI-R (Hoekstra et al. 2003) (Table 1). Therefore, we decided to use the present higher-order traits for reasons of parsimony with secondary analyses on facet level.

**Table 1:** Factor solution of principle components analysis with Varimax rotation on personality facet scores in the present study / compared to the original Dutch validation study (using all 30 facets)

Facet	Factor 1 neuroticism	Factor 2 extraversion	Factor 3 openness	Factor 4 conscientiousness	Cronbach’s *ɑ*
N1 anxiety	0.87/0.85				0.89/0.83
N3 depression	0.82/0.82				0.86/0.82
E2 gregariousness		0.77/0.75			0.77/0.76
E4 energy		0.73/0.53		–/0.49	0.67/0.66
E5 adventurism		0.62/0.61			0.68/0.63
O3 openness to feelings			0.58/0.64		0.70/0.75
O4 openness to change	−0.47/–		0.52/0.49		0.67/0.63
O5 openness to ideas			0.87/00.68		0.74/00.74
C1 efficacy	−0.41/			0.72/0.69	0.70/0.65
C2 orderliness				0.86/0.73	0.73/0.72
C5 self-discipline				0.70/0.81	0.78/0.79

Loadings < 0.40 not shown, – < 0.40 (unknown exact loading in the case of the original Dutch validation study by Hoekstra et al. 2003); Cronbach’s *ɑ* for the original Dutch validation study reflects the median of six studies

*C* conscientiousness, *E* extraversion, *N* neuroticism, *O* openness

Mood was measured using the Dutch short version of the Profile of Mood States (POMS) (McNair et al. 1971), consisting of 32 items (Wald and Mellenbergh 1990). These items assess five affective dimensions of which two are used here, which represent clinically the most relevant mood facets, anxiety (six items) and depression (eight items). Participants are asked to indicate “to what extent they felt that way lately.” The items are scored on five-point Likert scale ranging from 0 (not at all) to 4 (extremely). Internal consistencies of these subscales are good, ranging from Cronbach’s alpha of 0.87–0.91 (depression, in the present study being 0.92 at baseline) to 0.82–0.84 (anxiety, in the present study being 0.87 at baseline), and correlations with related and unrelated constructs showed convergent and discriminant validity (Wald and Mellenbergh 1990), and the subscales have been found to be sensitive to change over the course of a mindfulness-based psychological intervention (Haenen et al. 2016).

### Data Analyses

Data were analyzed using the IBM Statistical Package for the Social Sciences (SPSS) version 21.0. Baseline differences between the sample and specific reported facet-level norms for men and women from the general population as obtained from the Dutch Manual (Hoekstra et al. 2003) were examined by means of a one-sample *t* test. These analyses were sex specific as both mood and personality traits may differ considerably between the sexes (Hoekstra et al. 2003).

To test if scores on anxiety and depression changed over the three measurements, a linear mixed-model analysis was conducted, which takes into account participants with occasional missing values. The moderating effects of personality traits and socio-demographic variables on well-being were examined by including their interactions with time as relevant factors. The four main personality dimensions were used as continuous variables in the analyses. In addition, quartiles of the personality dimensions were formed to (i) enhance interpretability and (ii) allow for potential nonlinear effects. Additional factors in the model were time (baseline, post-intervention, and follow-up) and demographic and health-related variables including sex, age, education, job status, previous psychological treatment (yes versus no), and current use of psychotropic medication (yes versus no). In additional models, the 11 personality facets instead of the 4 domains were used as moderators to gain a more detailed picture of the role of individual personality facets. Because of the number of repeated tests, a Bonferroni correction of alpha level was applied in the facet analyses (by a factor of 2 or 3 as per domain, two or three facets were used), resulting in an alpha level of.025 for the neuroticism facets and 0.017 for the other facets.

## Results

Of the 173 participants of MBSR, 6 (3%) refused to participate in the study. Of the remaining 167 participants, 117 were women (70.1%), with a mean age of 45.8 years (SD 9.3). The majority had a higher professional education or university degree (*n* = 135, 80.9%) and held an occupation of at least 20 h per week (*n* = 132, 79%). Most participants (*n* = 124, 74.3%) were married or lived with a partner, and 48 (28.7%) have been treated for psychological problems of which 33 (19.8%) for depression.

Thirteen participants (7.5%) dropped out of MBSR (did not attend the last three sessions or more). When comparing dropouts with the remainder of the participants on demographic, and baseline mood and personality variables, dropouts appeared to be younger compared to treatment adherers (40.5 ± 8.6 versus 46.2 ± 9.2; *t*(165) = 2.15, *p* = 0.033). They also scored somewhat higher on openness (28.2 ± 3.2 versus 26.3 ± 3.1; *t*(165) = 2.11, *p* = 0.036), although on facet level, only trends were seen for openness to feelings (*p* = 0.062) and openness to ideas (*p* = 0.073) in the same direction. No other differences were found.

Ten (6%) participants did not complete the measurements after the MBSR program. At the 3-month follow-up, 18 participants (11%) had missing values. There were no differences between the participants not filling in the follow-up measurements and the adhering group regarding demographic variables or baseline mood and personality variables.

First, the baseline personality and mood scores of the present sample were compared to Dutch norms for women and men, as reported in the Dutch Manual (Hoekstra et al. 2003). This included a large sample, which was comparable to the present sample regarding most demographic characteristics, except education, about 80% of the current participants having higher education compared to about 50% of the norm sample. Compared to the facet-level norms, female participants scored higher on the neuroticism facets, and two of three openness facets, while they scored lower on all conscientiousness facets and on the extraversion facet of gregariousness (Table 2). Similar findings were obtained for men, although significant only for the neuroticism facets, openness for ideas, and self-discipline. Also regarding the POMS anxious and depressed mood, women and men had higher levels compared to the Dutch population (Table 2).

**Table 2:** Baseline personality and mood scores of the sample and the Dutch population for men and women

	Men (*n* = 50)					Women (*n* = 117)				
Variable	Participants		Population			Participants		Population		
	*μ*	*σ*	*μ*	*σ*	*p*	*μ*	*σ*	*μ*	*σ*	*p*
N1 anxiety	27.5	6.0	21.8	5.2	0.001	28.3	6.3	24.4	5.9	0.001
N3 depression	25.7	6.4	21.4	4.8	0.001	27.2	6.0	20.7	4.6	0.001
E2 gregariousness	23.7	4.3	24.4	5.4	0.274	23.5	5.4	25.6	5.5	0.001
E4 energy	25.5	4.2	25.5	4.3	1.0	26.3	4.3	25.5	4.3	0.040
E5 adventurism	23.1	5.0	23.2	5.0	0.887	20.6	4.8	21.5	5.0	0.056
O3 openness to feelings	28.5	4.6	27	4.1	0.021	30.0	4.0	29.1	4.0	0.019
O4 openness to change	22.8	3.4	22.3	4.6	0.308	23.5	4.7	22.3	4.6	0.007
O5 openness to ideas	28.0	5.1	25.4	5.4	0.001	26.0	4.8	24.8	4.8	0.008
C1 efficacy	27.4	3.7	28.4	3.5	0.067	26.3	4.2	28	3.3	0.001
C2 orderliness	25.6	4.4	26.2	4.2	0.371	25.4	4.8	26.7	4.3	0.006
C5 self-discipline	25.8	5.0	28.5	4.2	0.001	25.9	5.1	28.9	4.1	0.001
POMS										
Anxiety	11.1	4.8	3.6	4.0	0.001	11.1	5.7	5.1	4.9	0.001
Depression	10.0	7.2	1.9	4.4	0.001	9.8	7.2	2.6	4.5	0.001

*C* conscientiousness, *E* extraversion, *N* neuroticism, *O* openness, *POMS* Profile of Mood States

A basic mixed-model analysis with only time included as a factor showed a significant effect for both anxiety (*F*(2, 162) = 87.59, *p* < 0.001) and depression (*F*(2, 164) = 38.82, *p* < 0.001). After 8 weeks of MBSR intervention, anxiety lowered from an average of 11.10 (SD 5.44) to an average of 6.92 (SD 4.77) at post-intervention and 4.97 (SD 4.14) at follow-up. Similarly, depression decreased from an average of 9.83 (SD 7.18) to an average of 6.08 (SD 6.23) at the post-intervention and 4.91 (SD 5.54) at follow-up.

The first moderator analysis involved testing the effects of basic demographic and clinical variables on anxiety levels, both as main effects and as interactions with time: the variables sex, age, education, job status, previous psychological treatment, and use of psychotropic medication. The best model fit was found for the model including main and interaction with time effects reflecting moderation of the use of psychotropic medication (*F*(2, 156) = 3.48, *p* = 0.033) and age (*F*(2, 156) = 4.75, *p* = 0.010). Compared to the basic model fit improved to −2 log likelihood = 1019.9, change *χ* ^2^ (3) = 36.2, *p* < 0.0001, AIC = 1049.9, and BIC = 1112.0. Participants on psychotropic medication showed larger decreases in anxiety between post-intervention and follow-up but not between pre- and post-intervention, compared to participants not on medication. Older participants showed smaller decreases in anxiety over time.

In the analysis with depression as the outcome variable, no moderation effects by demographic variables were found. Because of the effects on anxiety, in subsequent analyses, age and psychotropic medication were included with their main and interaction effects.

In the next step, all four personality dimensions were included. For both depressive and anxiety symptoms, however, model fit improved most when only neuroticism and its interaction with time was included in the model, for depression, −2 log likelihood = 901.6, change *χ* ^2^ (3) = 115.6, *p* < 0.0001, AIC = 937.6, and BIC = 1012.2, and for anxiety, 2 log likelihood = 896.0, change *χ* ^2^ (3) = 123.9, *p* < 0.0001, AIC = 932.0, and BIC = 1006.6. The other personality dimensions did not show any significant effects, which was also the case when entered separately into the model.

**Fig. 1:**
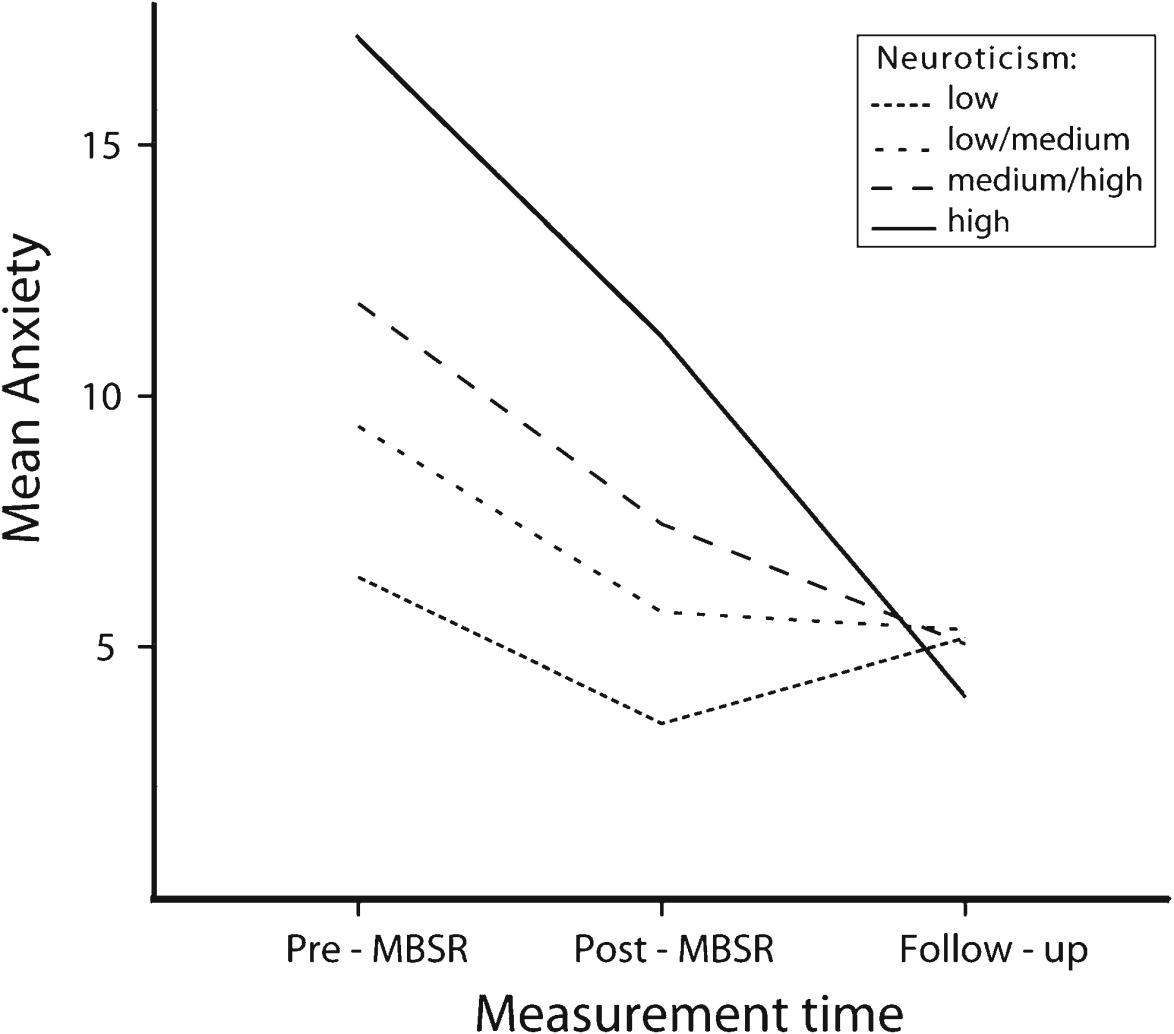
Estimated marginal means of anxiety over three time points per quartile of neuroticism

For anxiety, in this model including neuroticism, the effects of medication and age were no longer significant (*p* > 0.10). Besides a main effect of neuroticism (*F*(1, 166) = 118.38, *p* < 0.001), reflecting higher anxiety with higher neuroticism, the time × neuroticism interaction was significant (*F*(2, 161) = 31.01, *p* < 0.001). Participants higher in neuroticism showed stronger decreases in anxiety, as evidenced in the illustrative analysis based on quartiles of neuroticism (Table 3 and Fig. 1).

**Table 3:** Estimates of mean difference in anxiety symptoms between pre-intervention, post-intervention, and follow-up measurements for each of the four quartiles of neuroticism

Neuroticism quartile	Pre-MBSR (T1), *M* (SE)	Post-MBSR (T2), *M* (SE)	Follow-up (T3), *M* (SE)	Difference (T1–T3)	Cohen’s *d*
Low	5.30 (0.70)	2.50 (0.68)	1.59 (0.67)	3.71	0.42
Low-medium	8.90 (0.71)	4.83 (0.71)	1.47 (0.69)	7.43	0.82
Medium-high	10.33 (0.63)	6.36 (0.63)	2.22 (0.60)	8.10	1.02
High	14.96 (0.64)	9.25 (0.63)	1.24 (0.63)	13.72	1.67

Model includes socio-demographic variables and the four personality dimensions

However, because neuroticism was strongly associated with anxiety at baseline (*r* = 0.70), analyses should be corrected for this confounding (i.e., individuals reporting higher levels of distress have more potential for improvement). Because one cannot control for the baseline levels of a variable in an analysis in which the same baseline is also included as dependent variable, and because the main aim of the study was to examine moderating effects on decrease in anxiety and depressive mood, for these analyses, we used change scores from pre- to post-intervention and from post-intervention to follow-up as outcomes in the mixed-model analysis including the same variables as predictors as above complemented by the main effect of baseline anxiety and its interaction with time. Such procedure is well defendable as change scores provide an unbiased estimate of true change (Rogosa 1988), which is our main focus.

**Fig. 2:**
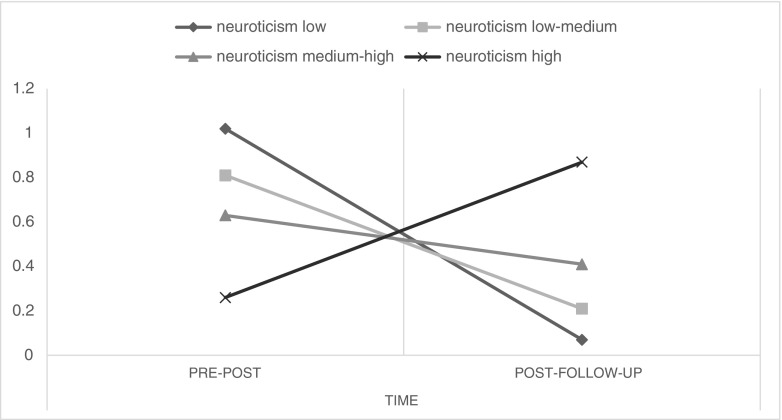
The amount of anxiety reduction from pre- to post-intervention and from post-intervention to follow-up across quartiles of neuroticism, controlled for baseline anxiety levels

In this analysis, baseline anxiety was the strongest predictor of overall change in anxiety as shown by its main effect (*F*(1, 132) = 140.12, *p* < 0.001; estimate = 0.37, 95% CI = 0.14–0.60). Its interaction with time just did not reach the conventional level of significance (*F*(1, 150) = 3.87, *p* = 0.051), but the interaction of time × neuroticism did (*F*(1, 148) = 12.53, *p* = 0.001). This interaction reflected that higher neuroticism was associated with a smaller decrease in anxiety from pre- to post-intervention but also a larger decrease in anxiety after the intervention ended. This is illustrated in an analysis based on quartiles instead of continuous scores of neuroticism (interaction with time being significant at *p* = 0.005), the decrease from pre- to post-intervention for the lowest through highest neuroticism quartile being 1.0, 0.8, 0.6, and 0.3 and from post-intervention to follow-up being 0.1, 0.2, 0.4, and 0.9, respectively. Thus, the overall decrease from baseline to the 3-month follow-up was similar for the groups (Fig. 2).

**Fig. 3:**
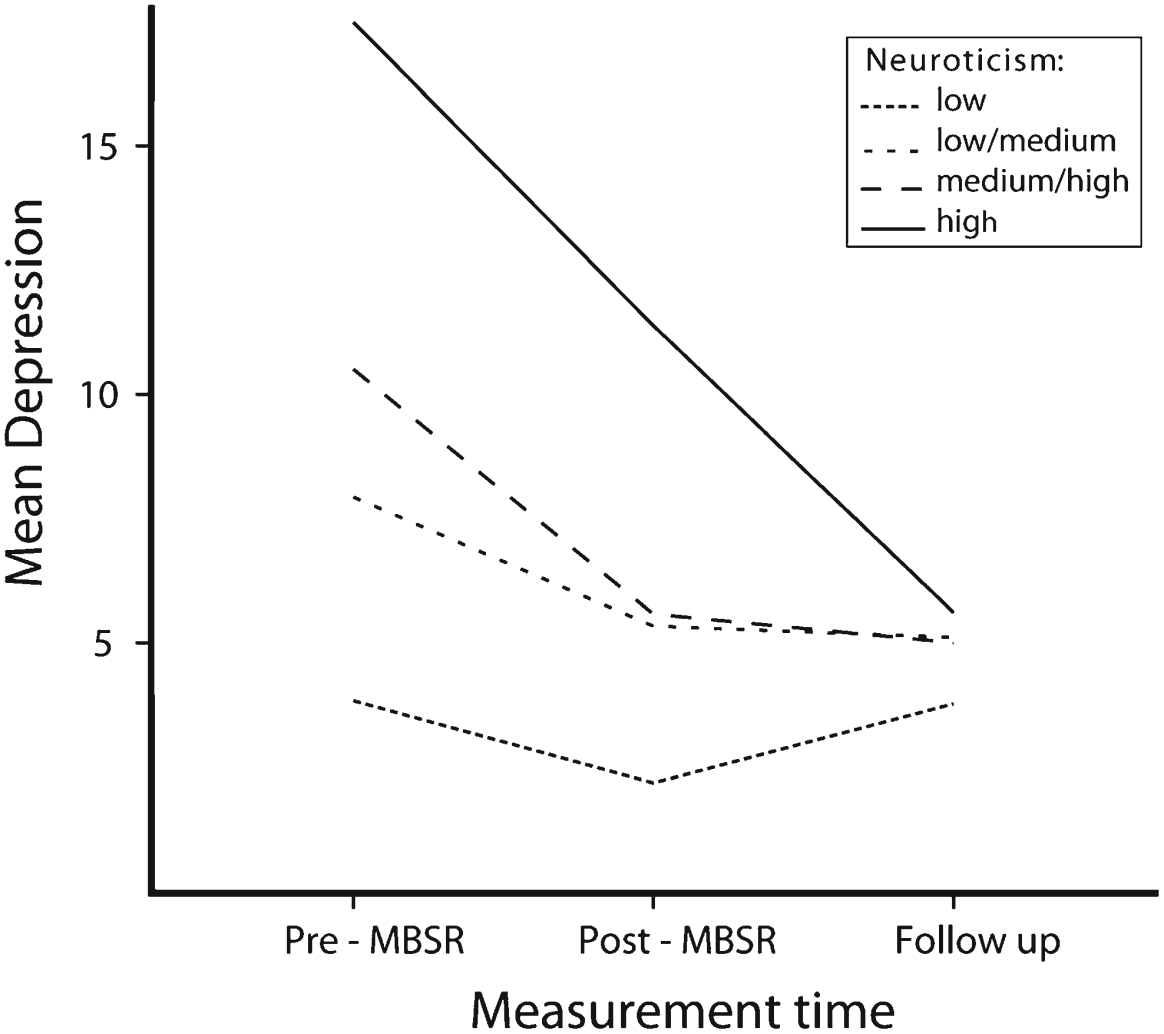
Estimated marginal means of depression over three time points per quartile of neuroticism

In the regular mixed-model analysis on depressive mood, also a main effect of neuroticism (*F*(1, 164) = 135.73, *p* < 0.001) and a time × neuroticism interaction effects appeared (*F*(2, 160) = 18.19, *p* < 0.001). Again, effects reflected that participants higher in neuroticism showed a stronger decrease in depression, as can be best seen in the analysis based on quartiles of neuroticism (Table 4 and Fig. 3). Again, to control for baseline depressive mood (correlating *r* = 0.68 with neuroticism), a similar analysis as above on change scores was performed. Again, a main strong effect of baseline depression appeared (*F*(1, 132) = 174.96, *p* < 0.001), showing a positive association with decrease in depressive symptom across time (estimate = 0.56, 95% CI = 0.33–0.79). In addition, a main effect of neuroticism appeared (*F*(1, 132) = 8.18, *p* = 0.005), but the coefficient of its effect did not reach significance (estimate = −0.01, 95% CI = −0.05–0.02). Also when using quartiles of neuroticism, no significant main or interaction effects including neuroticism emerged (*p* > 0.10), suggesting lack of robustness of the effect.

**Table 4:** Estimates of mean difference in depression symptoms between pre-intervention, post-intervention, and follow-up measurements for each of the four quartiles of neuroticism

Neuroticism quartile	Pre-MBSR (T1), *M* (SE)	Post-MBSR (T2), *M* (SE)	Follow-up (T3), *M* (SE)	MD (T1–T3)	Cohen’s *d*
Low	4.75 (1.03)	4.37 (1.11)	2.80 (0.91)	1.96	0.16
Low-medium	10.43 (1.0)	7.53 (1.12)	2.96 (0.95)	7.47	0.60
Medium-high	11.84 (0.88)	8.03 (0.96)	5.02 (0.82)	6.82	0.62
High	18.10 (0.88)	12.20 (0.94)	4.42 (0.85)	13.68	1.23

Model includes socio-demographic variables and the four personality dimensions

When examining the moderating effects of personality in greater detail on facet level, by including the individual facets instead of the basic dimensions, controlling for the effects of age and psychotropic medication, none of the facets, except the neuroticism facets, showed significant effects. Only the neuroticism facets showed effects highly similar to the effects described above on trait level. Specifically, changes in anxiety levels were predicted by the neuroticism-anxiety facet, as reflected by its interaction with time (*F*(2, 159) = 15.83, *p* < 0.001), while changes in depression levels were predicted by the neuroticism-depression facet (*F*(2, 158) = 8.44, *p* < 0.001). Only for anxiety, this effect remained significant when the effects were adjusted for baseline anxiety symptoms using change scores as outcome (*F*(1, 150) = 15.35, *p* < 0.001), not for depression (*p* > 0.10).

## Discussion

The aim of the present study was to examine potentially moderating effects of relevant personality factors and basic socio-demographic variables regarding changes in anxious and depressed mood during and after a mindfulness-based stress reduction intervention. The identification of such possible moderators might help differentiating people for whom this type of intervention is beneficial from those for whom it is not.

First, consistent with previous studies as summarized in recent reviews and meta-analyses (Fjorback et al. 2011; Hofmann et al. 2010; Khoury et al. 2013), overall decreasing anxious and depressed mood have been found in the present study. Importantly, this positive effect was not only visible immediately after the intervention, but anxiety and depression continued to decrease at the 3-month follow-up, showing relatively lasting changes in psychological well-being with overall a large effect size from pre-intervention to follow-up.

Regarding the question of moderators of distress reduction, the first analysis including only demographic and mental health indicators showed that age and use of psychotropic medication modulated the decrease. However, these effects disappeared when personality was introduced into the models. Most personality domains did not show significant moderator effects, except neuroticism. Results of the initial analysis showed larger benefit with higher neuroticism on both anxiety and depressive mood. These findings are in line with the previous study conducted in students (de Vibe et al. 2015). However, this effect seems to have been largely confounded by baseline mood problems; the higher the baseline levels are, the stronger the reduction is. This effect is well known in the literature and may be a consequence of a larger potential to change when scores are more extreme or a regression to the mean effect. When baseline levels were controlled, only one effect remained; higher neuroticism was associated with a smaller decrease in anxiety at post-intervention but a larger decrease between post-intervention and the 3-month follow-up. In other words, while overall change to follow-up was not substantially different, the decrease in anxiety was delayed in people scoring higher on neuroticism.

These findings may be important when comparing them with other interventions for psychological stress and depression complaints, notably cognitive behavior therapy (CBT). In general, CBT shows weaker benefit regarding anxious and depressed mood for those high in neuroticism, perhaps making change more difficult for those having a more stable propensity to negative affect (Quilty et al. 2008; Spek et al. 2008; Taylor and Mclean 1993; Wolitzky-Taylor et al. 2012). One may speculate about the possibility that when such a stable propensity towards negative affectivity is present, mindfulness may be more effective, albeit not immediately. The present finding on anxiety seem to suggest that a mindfulness-based intervention may yield a cognitive change in people high in neuroticism, which may be unique to mindfulness and which makes them decrease in anxiety, although not immediately.

Such cognitive processes may include mindfulness skills which people high in neuroticism show in lower levels compared to emotionally more stable individuals, i.e., acceptance, acting with awareness, and being nonreactive to one’s disturbing thoughts (Baer et al. 2004; Baer et al. 2006; Brown and Ryan 2003; Hanley 2016). Also, as these individuals have a tendency to ruminate (Costa and McCrae 1992) and to avoid experiences (Lommen et al. 2010), both being processes that diminish as a result of mindfulness (Hayes and Feldman 2004; Nyklíček 2011; Shapiro et al. 2006), these processes seem promising as potential mechanisms involved in the beneficial effects of mindfulness, especially in individuals scoring high on neuroticism. The fact that the benefit was shown only after the intervention may not be surprising as one can imagine that it takes time, especially for individuals showing high neuroticism, to be able to make such a fundamental cognitive-emotional shift from high emotional reactivity and/or avoidant behavior to acceptance, nonreactivity, and/or decentering from ruminative thought (Kabat-Zinn 1990).

In line with this interpretation, a recent study has found that MBSR decreased the trait of negative affectivity, which was interpreted as possibly reflecting the ability of mindfulness of changing basic stable propensities by its radically different approach to one’s own psychological processes (Nyklíček et al. 2013). Interestingly, in one recent direct comparison study between CBT and MBSR for anxiety disorders, MBSR outperformed CBT in those patients with comorbid moderate to severe depressive symptoms (Arch and Ayers 2013), which are known to be strongly associated with the traits of negative affectivity and neuroticism.

Regarding extraversion, the present results contrast previous findings. While a study on a CBT program found more benefit in people scoring higher on extraversion (Quilty et al. 2008), a study in MBCT in people with diabetes reported lower benefit in participants scoring high on extraversion (Nyklíček et al. 2016). The present study showed no effects of this trait. Perhaps the central mindfulness aspect of (self) acceptance in combination with a safe and accepting group format of the intervention largely focusing on introspective and interoceptive processes may be well fitting the more introverted participants, which may have balanced out the expected potential benefit of extraversion associated with larger psychological flexibility (Kashdan and Rottenberg 2010). This suggestion seems in line with the results of the MBCT study in diabetes patients, which showed not only lower decrease in symptoms of anxiety and depression but also a substantially higher dropout in people scoring high on extraversion (Nyklíček et al. 2016). Our results are in line with those of a study in a student sample, in which extraversion did not seem to moderate the effects of a mindfulness training (de Vibe et al. 2015). A potentially relevant difference between the studies is that in contrast to research not finding an effect of extraversion, the MBCT study in diabetes did not use the words mindfulness or meditation during recruitment of participants, instead referring to learning attention regulation skills (van Son et al. 2013). This might have increased the subscription of participants who would otherwise not subscribe, revealing a potential misfit with the intervention only during participation in that study.

Also, conscientiousness did not show moderating effects. These were hypothesized as being the result of more dedicated practice in the more conscientious people, which has been claimed to be important for obtaining benefit (Kabat-Zinn 1990). However, previous studies have reported inconsistent findings regarding the correlation between the amount of home practice and the extent of decrease in psychological symptoms (Nyklíček et al. 2013).

For openness, also no effects were obtained, which was not hypothesized as higher scores were expected to be beneficial. Perhaps a relatively high score in openness for feelings and new ideas may already be present in most people participating in a mindfulness intervention, reflecting a selection bias. Indeed, participants in the present study scored significantly higher on various openness facets compared to the Dutch population.

Dropout was quite low in this sample. The somewhat higher score on openness in dropouts compared to adherers is surprising as, if anything, the opposite may have been expected. Openness to feelings and new ideas may be regarded as likely facilitators of the training process during a psychological intervention based on a new approach, such as mindfulness, in which openness to feelings is an important attitude. We do not have a satisfactory explanation for this finding, except speculating that this finding may have been due to chance as on facet level, the differences were not significant.

To put the results into context, we also examined if the people who participated in MBSR differed from the general Dutch population. In this study, the participants had higher levels of neuroticism than the average Dutch population with a medium-to-large effect size. This difference was to be expected, since it is known that neuroticism strongly predisposes to experience distress for which participants in the present study sought help by subscribing to the MBSR intervention. Further, both sexes scored lower on the conscientiousness facet self-discipline, and women only also on efficacy and order, although effect sizes of the latter two were low. Also, generally low effect sizes were found for the differences regarding the extraversion facet of gregariousness (lower in female participants compared to population) and openness (higher in participants of both sexes). The effect of openness may also have been expected as discussed above.

It may be noted that while subscribing to a mindfulness-based intervention seems to be associated with a generally less adaptive personality trait profile, mindfulness as a trait is associated with adaptive scores on personality traits, i.e., especially lower scores on neuroticism and higher on conscientiousness (Baer et al. 2004; Brown and Ryan 2003; Van den Hurk et al. 2011). This may put forward the hypothesis that learning mindfulness may change scores on personality dimensions. Recently, some evidence for this position has been provided by two studies. A randomized trial showed MBSR to be associated with a decrease on the traits of negative affectivity (strongly associated with neuroticism) and social inhibition (Nyklíček et al. 2013). In another recent study, positive changes were reported regarding the character facets of intrapersonal (self-directedness), interpersonal (cooperativeness), and transpersonal (self-transcendence) levels of the Temperament and Character Inventory (Campanella et al. 2014).

## Limitations

The following limitations are acknowledged. First and foremost, the lack of a control group does not permit conclusions regarding the uniqueness of MBSR regarding the moderating effects obtained. It cannot be excluded that these effects may also have been found in other interventions or even are due to just a differential regression to the mean effect. However, this does not seem likely in light of the fact that some of the effects seem to be different from effects obtained in CBT. Nevertheless, future studies should include a well-defined control group to test the specificity of the effects. In addition, the fact that people subscribed themselves to MBSR potentially introduced a self-selection bias not permitting generalization of the results to non-self-selected samples. Also, the fact that majority of the participants consisted of well-educated women limits generalizability to other groups. The lack of data regarding adherence is a limitation as adherence may be related to both personality and treatment outcome. Finally, assessing changes in mindfulness skills may have shed some light on potential mechanisms involved in the moderation effects. Future research should address this issue.

## References

[CR1] Arch JJ, Ayers CR (2013). Which treatment worked better for whom? Moderators of group cognitive behavioral therapy versus adapted mindfulness based stress reduction for anxiety disorders. Behaviour Research and Therapy.

[CR2] Baer RA (2009). Self-focused attention and mechanisms of change in mindfulness-based treatment. Cognitive Behaviour Therapy.

[CR3] Baer RA, Smith GT, Allen KB (2004). Assessment of mindfulness by self-report: the Kentucky inventory of mindfulness skills. Assessment.

[CR4] Baer RA, Smith GT, Hopkins J, Krietemeyer J, Toney L (2006). Using self-report assessment methods to explore facets of mindfulness. Assessment.

[CR5] Bohlmeijer E, ten Klooster PM, Fledderus M, Veehof M, Baer R (2011). Psychometric properties of the five facet mindfulness questionnaire in depressed adults and development of a short form. Assessment.

[CR6] Brown KW, Ryan RM (2003). The benefits of being present: mindfulness and its role in psychological well-being. Journal of Personality and Social Psychology.

[CR7] Campanella F, Crescentini C, Urgesi C, Fabbro F (2014). Mindfulness-oriented meditation improves self-related character scales in healthy individuals. Comprehensive Psychiatry.

[CR8] Carlson KD, Herdman AO (2012). Understanding the impact of convergent validity on research results. Organizational Research Methods.

[CR9] Chambers R, Gullone E, Allen NB (2009). Mindful emotion regulation: an integrative review. Clinical Psychology Review.

[CR10] Chiesa A, Serretti A (2010). A systematic review of neurobiological and clinical features of mindfulness meditations. Psychological Medicine.

[CR11] Costa, P. T., & McCrae, R. R. (1992). *Revised NEO Personality Inventory (NEO-PI-R) and the Five Factor Inventory (NEO-FFI): Professional manual*. Odessa, FL: Psychological Assessment Resources.

[CR12] de Vibe M, Solhaug I, Tyssen R, Friborg O, Rosenvinge JH, Sorlie T (2015). Does personality moderate the effects of mindfulness training for medical and psychology students?. Mindfulness.

[CR13] Fjorback LO, Arendt M, Ornbol E, Fink P, Walach H (2011). Mindfulness-based stress reduction and mindfulness-based cognitive therapy: a systematic review of randomized controlled trials. Acta Psychiatrica Scandinavica.

[CR14] Giluk TL (2009). Mindfulness, big five personality, and affect: a meta-analysis. Personality and Individual Differences.

[CR15] Haenen S, Nyklíček I, Van Son J, Pop V, Pouwer F (2016). Mindfulness facets as differential mediators of short and long-term effects of mindfulness-based cognitive therapy in diabetes outpatients: findings from the DiaMind randomized trial. Journal of Psychosomatic Research.

[CR16] Hanley AW (2016). The mindful personality: associations between dispositional mindfulness and the five factor model of personality. Personality and Individual Differences.

[CR17] Hayes AM, Feldman G (2004). Clarifying the construct of mindfulness in the context of emotion regulation and the process of change in therapy. Clinical Psychology: Science and Practice.

[CR18] Hoekstra, H. A., Ormel, J., & de Fruyt, F. (1996). NEO persoonlijkheids vragenlijsten: NEO-PI-R; NEO-FFI [NEO personality questionnaires: NEO-PI-R; NEO-FFI]. Lisse, Netherlands: Swets.

[CR19] Hoekstra, H. A., Ormel, J., & De Fruyt, F. (2003). Handleiding NEO persoonlijkheidsvragenlijsten NEO-PI-R en NEO-FFI [Manual NEO personality questionnaires NEO-PI-R and NEO-FFI] Lisse: Harcourt.

[CR20] Hofmann SG, Sawyer AT, Witt AA, Oh D (2010). The effect of mindfulness-based therapy on anxiety and depression: a meta-analytic review. Journal of Consulting and Clinical Psychology.

[CR21] Hollis-Walker L, Colosimo K (2011). Mindfulness, self-compassion, and happiness in non-meditators: a theoretical and empirical examination. Personality and Individual Differences.

[CR22] Kabat-Zinn, J. (1990). Full catastrophe living: using the wisdom of your body and mind to face stress, pain, and illness. New York: Delacourt.

[CR23] Kabat-Zinn J, Massion AO, Kristeller J, Peterson LG, Fletcher KE, Pbert L (1992). Effectiveness of a meditation-based stress reduction program in the treatment of anxiety disorders. American Journal of Psychiatry.

[CR24] Kashdan TB, Rottenberg J (2010). Psychological flexibility as a fundamental aspect of health. Clinical Psychology Review.

[CR25] Keng SL, Smoski MJ, Robins CJ (2011). Effects of mindfulness on psychological health: a review of empirical studies. Clinical Psychology Review.

[CR26] Khoury B, Lecomte T, Fortin G, Masse M, Therien P, Bouchard V (2013). Mindfulness-based therapy: a comprehensive meta-analysis. Clinical Psychology Review.

[CR27] Kokkonen M, Pulkkinen L (2001). Examination of the paths between personality, current mood, its evaluation, and emotion regulation. European Journal of Personality.

[CR28] Latzman RD, Masuda A (2013). Examining mindfulness and psychological inflexibility within the framework of big five personality. Personality and Individual Differences.

[CR29] Lommen MJJ, Engelhard IM, van den Hout MA (2010). Neuroticism and avoidance of ambiguous stimuli: better safe than sorry?. Personality and Individual Differences.

[CR30] McNair, D. M., Lorr, M., & Droppelman, L. F. (1971*). Profile of Mood States (POMS) Manual. San Diego*, *C*A: Educational and Industrial Testing Service.

[CR31] Nyklíček I, Nyklíček I, Vingerhoets A, Zeelenberg M (2011). Mindfulness, emotion regulation, and well-being. Emotion regulation and well-being.

[CR32] Nyklíček I, van Beugen S, Denollet J (2013). Effects of mindfulness-based stress reduction on distressed (type D) personality traits: a randomized controlled trial. Journal of Behavioral Medicine.

[CR33] Nyklíček I, van Son J, Pop VJ, Denollet J, Pouwer F (2016). Does mindfulness-based cognitive therapy benefit all people with diabetes and comorbid emotional complaints equally? Moderators in the DiaMind trial. Journal of Psychosomatic Research.

[CR34] Poon A, Danoff-Burg S (2011). Mindfulness as a moderator in expressive writing. Journal of Clinical Psychology.

[CR35] Quilty LC, De Fruyt F, Rolland JP, Kennedy SH, Rouillon PF, Bagby RM (2008). Dimensional personality traits and treatment outcome in patients with major depressive disorder. Journal of Affective Disorders.

[CR36] Rogosa D, Schaie KW, Campbell RT, Meredith WM, Rawlings SC (1988). Myths about longitudinal research. Methodological issues in aging research.

[CR37] Segal, Z., Williams, J. M., & Teasdale, J. D. (2002). *Mindfulness-Based Cognitive Therapy for depression*. New York: Guilford.

[CR38] Shapiro SL, Brown KW, Thoresen C, Plante TG (2011). The moderation of mindfulness-based stress reduction effects by trait mindfulness: results from a randomized controlled trial. Journal of Clinical Psychology.

[CR39] Shapiro SL, Carlson LE, Astin JA, Freedman B (2006). Mechanisms of mindfulness. Journal of Clinical Psychology.

[CR40] Spek V, Nyklíček I, Cuijpers P, Pop V (2008). Predictors of outcome of group and internet-based cognitive behavior therapy. Journal of Affective Disorders.

[CR41] Taylor S, Mclean P (1993). Outcome profiles in the treatment of unipolar depression. Behaviour Research and Therapy.

[CR42] Trapnell PD, Campbell JD (1999). Private self-consciousness and the five-factor model of personality: distinguishing rumination from reflection. Journal of Personality and Social Psychology.

[CR43] Van den Hurk PAM, Wingens T, Giommi F, Barendregt HP, Speckens AEM, Van Schie HT (2011). On the relationship between the practice of mindfulness meditation and personality: an exploratory analysis of the mediating role of mindfulness skills. Mindfulness.

[CR44] van der Velden AM, Kuyken W, Wattar U, Crane C, Pallesen KJ, Dahlgaard J, Piet J (2015). A systematic review of mechanisms of change in mindfulness-based cognitive therapy in the treatment of recurrent major depressive disorder. Clinical Psychology Review.

[CR45] van Son J, Nyklíček I, Pop VJ, Blonk MC, Erdtsieck RJ, Spooren PF (2013). The effects of a mindfulness-based intervention on emotional distress, quality of life, and HbA1c in outpatients with diabetes (DiaMind): a randomized controlled trial. Diabetes Care.

[CR46] Wald FDM, Mellenbergh GJ (1990). De verkorte versie van de Nederlandse vertaling van de Profile of Mood States (POMS) [the shortened version of the Dutch translation of the Profile of Mood States (POMS)]. Nederlands Tijdschrift voor de Psychologie.

[CR47] Wolitzky-Taylor KB, Arch JJ, Rosenfield D, Craske MG (2012). Moderators and non-specific predictors of treatment outcome for anxiety disorders: a comparison of cognitive behavioral therapy to acceptance and commitment therapy. Journal of Consulting and Clinical Psychology.

